# Supplementary material for: Stability Program in Dendritic Cell Vaccines: A “Real-World” Experience in the Immuno-Gene Therapy Factory of Romagna Cancer Center
Source: Vaccines (Basel). 2022 Jun 23;10(7):999. doi: 10.3390/vaccines10070999 (PMC9323699; doi:10.3390/vaccines10070999)
Supplement: Supplementary file 1 [file vaccines-10-00999-s001.zip › vaccines-1746837-supplementary.pdf]

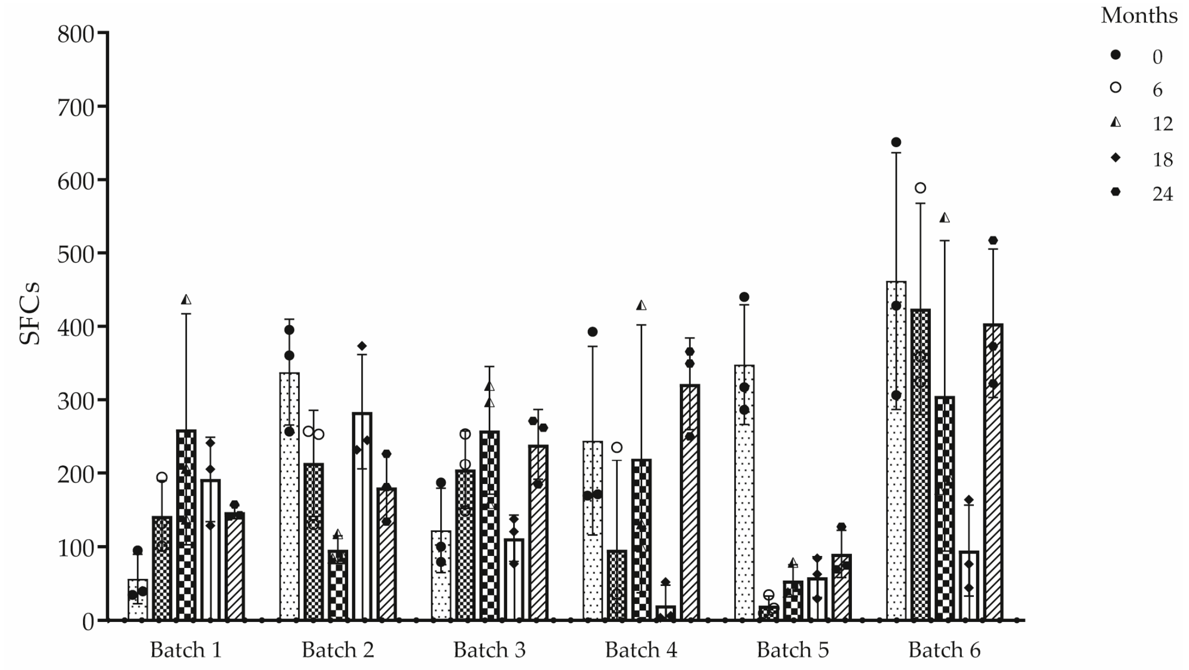

Figure S1. Results of ELISPOT Costim assay are shown as a bar-plot of SFCs (mean ± SD) at several time points. The different symbols shapes represent the mean of quadruplicate of each activated CD3+ T cell donor.

Table S1. Number of Spot Forming Cells (SFCs) of activated CD3+ T cells (3 donor) by dendritic cells inpresence of 0.02 µg/ml OKT3 antibody.

**Batch Number**	**Month**	**Donor 1 CD3+ T-cells**				**Donor 2 CD3+ T-cells**				**Donor 3 CD3+ T-cells**			
	0	17	24	40	57	44	45	34	35	121	115	80	63
	6	62	137	104	98	179	169	205	223	196	162	93	75
1	12	169	194	227	233	430	467	434	417	175	159	116	98
	18	170	251	216	328	201	220	220	180	156	138	127	93
	24	134	137	146	147	135	173	118	145	219	159	140	110
	0	321	347	389	385	417	387	410	367	265	236	263	264
	6	95	126	173	126	233	286	264	230	288	253	250	238
2	12	131	113	84	142	99	63	83	92	104	83	89	70
	18	193	241	250	296	383	358	380	373	259	267	228	174
	24	209	242	252	203	171	155	185	211	157	139	129	111
	0	136	165	199	248	63	25	107	123	171	121	74	34
	6	175	221	208	243	247	238	296	233	131	158	172	133
3	12	214	290	277	407	399	395	245	238	169	183	168	114
	18	105	139	151	155	85	97	61	64	123	125	140	93
	24	242	250	317	276	254	230	274	290	234	186	177	140
	0	352	430	388	400	216	126	163	172	174	192	156	162
	6	200	254	239	249	14	24	10	20	32	48	37	26
4	12	410	435	432	440	102	89	148	184	138	93	87	79
	18	1	5	4	4	6	5	5	8	63	44	44	57
	24	325	376	334	362	348	296	417	402	269	268	256	208
	0	278	337	283	248	286	316	333	333	427	499	423	411

	6	5	5	16	13	2	33	17	14	54	31	30	24
5	12	60	95	85	75	57	39	42	44	41	23	39	49
	18	69	61	61	60	24	31	29	34	67	114	69	86
	24	63	89	63	62	140	116	134	118	101	84	58	55
	0	360	448	460	444	351	277	283	314	675	676	670	584
	6	426	420	427	24	309	410	366	349	518	636	587	615
6	12	109	172	206	222	168	240	141	209	581	541	538	536
	18	195	332	81	46	86	61	80	79	59	54	41	23
	24	194	359	431	306	433	457	598	580	410	444	224	413
